# Time Scales of CD4^+^ T Cell Depletion in HIV Infection

**DOI:** 10.1371/journal.pmed.0040193

**Published:** 2007-05-22

**Authors:** Rob J De Boer

## Abstract

Rob De Boer discusses a new study that investigated whether a runaway process of T cell activation/infection would be compatible with the slow time scale of memory CD4^+^ T cell depletion in humans with chronic HIV infection.

The hallmark of HIV infection is the depletion of CD4^+^ T cells in peripheral blood, lymphoid organs, and mucosal tissues [1]. Since CD4^+^ T cells play an essential role in immune defenses against almost all pathogens, HIV-positive patients are subject to a variety of opportunistic infections. Despite decades of intensive research, the mechanisms underlying CD4^+^ T cell depletion remain widely debated. CD4^+^ T cells constitute the major target cells for HIV. David Ho and colleagues initially proposed that CD4^+^ T cells disappear by viral infection and subsequent cytolytic effects, and/or by the removal of infected CD4^+^ T cells by the immune response [2]. To account for the slow time scale of CD4^+^ T cell depletion, they suggested the “tap and drain” hypothesis [2], according to which CD4^+^ T cell production (a wide-open tap) is ultimately exhausted by the homeostatic response that is almost perfectly compensating for the large daily loss (the drain) of CD4^+^ T cells due to HIV infection.

More recently the “immune activation” hypothesis has gained popularity [3]. HIV infection in humans, and simian immunodeficiency virus (SIV) infection in rhesus macaques, is characterized by increased rates of cell division in CD4^+^ and CD8^+^ T cells, natural killer cells, and B cells [4], and by up-regulation of various activation markers [5]. Most strikingly, Sooty Mangabeys and African Green monkeys that become naturally infected with their own strain of SIV also have high viral loads, but have hardly any disease progression to AIDS, presumably because they maintain almost normal activation levels of their CD4^+^ and CD8^+^ T cells [6].

**Linked Research Article**

This Perspective discusses the following new study published in *PLoS Medicine*:

Yates A, Stark J, Klein N, Antia R, Callard R (2007) Understanding the slow depletion of memory CD4^+^ T cells in HIV infection. PLoS Med 4(5): e177. doi:10.1371/journal.pmed.0040177

Using a simple mathematical model, Andrew Yates and colleagues show that a runaway cycle of T cell activation and infection cannot explain the slow rate of CD4^+^ cell decline during chronic HIV infection.

## A New Study

The apparently advantageous absence of immune activation in the host–pathogen relations that have coevolved over many generations raises the question, “Why would the immune system be so prominently activated in humans and macaques?” An intriguing possibility is that the virus has evolved strategies to increase the availability of suitable target cells by activating CD4^+^ T cells. Increasing target cell availability would be like “fueling the fire,” resulting in more infection and runaway depletion of CD4^+^ T cells.

In a new study published in *PLoS Medicine*, Andrew Yates and his colleagues investigate whether such a runaway process would be compatible with the slow time scale of memory CD4^+^ T cell depletion in humans [7]. Immune activation is not automatically an explanation for T cell depletion. Increasing cell division rates of memory T cells by immune activation could also increase their capacity of self-renewal, and lead to increased cell counts. To explain “depletion by activation,” Yates et al. propose that immune activated CD4^+^ T cells have a very short life span, which means that the immune-activated cells are lost by activation-induced cell death [7].

## Too Rapid Depletion

The authors show that this model of immune activation readily explains the depletion of CD4^+^ T cells, and that the level to which cell counts are depleted depends on the immune activation rate. The depletion goes much too fast, however. Once immune activation is turned on, the CD4^+^ T cell counts approach a new low “set point” on a time scale that is largely determined by the average time between homeostatic divisions—i.e., the time scale of T cell homeostasis. Estimates for the lifespan of memory T cells vary considerably, but are typically estimated to have a time scale of months [8]. Depletion by immune activation should therefore be completed in a few months. This time scale is unrealistically fast, given the clinical course of HIV infection: the challenge of explaining CD4^+^ T cell depletion in chronic HIV infection is its slow time scale of about a decade. Thus, Yates and colleagues have rejected the “runaway” hypothesis [7].

## Simple or Complicated Mathematical Models

The authors rejected this hypothesis on the basis of various simple mathematical models. How do we know that these models are correct, and that they indeed allow one to reject an immunological hypothesis? This is a difficult question, and some researchers have argued that caricature models (i.e., models implementing only the most essential processes) are just too simple and unrealistic to have any relevance for immunological research. Others argue that models should remain simple because then they are excellent tools that allow one “to think clearly” [9]. Yates and colleagues' study [7] in fact uses models as “thought experiments” to test the dynamic consequences of immune activation. Because their models are simple and natural, it is perfectly understandable that they would find that an immune activation model depletes CD4^+^ T cells too rapidly for what is seen clinically. In retrospect, it seems a very natural result, increasing our insights on the dynamic effects of immune activation.

## Slow Time Scale

Apparently we need a truly slow process to drive the slow depletion of CD4^+^ T cells, and Yates et al. discuss a number of candidate models [7]. Because naïve T cells operate on very slow time scales, they are a major suspect. Recent studies with long-term deuterium labeling in healthy volunteers suggest that naïve T cells have lifespans of several years [8]. Similar labeling techniques in HIV-positive patients show markedly increased turnover rates of naïve T cells ([10] and Nienke Vrisekoop et al., unpublished data], which is in good agreement with the dilution of T cell receptor excision circles seen in HIV-positive patients [11]. Both CD4^+^ and CD8^+^ naïve T cells are gradually depleted during HIV infection [12], and increased age is an important risk factor for HIV-1 disease progression [13]. Whether or not slow depletion of naïve T cells affects the depletion rates of memory T cells, however, remains an open question. As yet, we have a very limited understanding of the maintenance mechanisms of effector/ memory T cells during rampant chronic viral infection, calling for more research into the population dynamics of the immune system.

## Abbreviations

SIV: simian immunodeficiency virus
